# Mouse Systems to Model Hepatitis C Virus Treatment and Associated Resistance

**DOI:** 10.3390/v8060176

**Published:** 2016-06-22

**Authors:** Ahmed Atef Mesalam, Koen Vercauteren, Philip Meuleman

**Affiliations:** 1 Center for Vaccinology, Department of Clinical Chemistry, Microbiology and Immunology, Ghent University, Ghent 9000, Belgium; ahmed.mesalam@ugent.be (A.A.M.); koen.vercauteren@ugent.be (K.V.); 2 Therapeutic Chemistry Department, National Research Centre (NRC), Dokki, Cairo 12622, Egypt; 3 Laboratory of Virology and Infectious Disease, The Rockefeller University, New York, NY 10065, USA

**Keywords:** HCV, animal models, therapy, direct acting antiviral agents, humanized mice, resistance, deep sequencing

## Abstract

While addition of the first-approved protease inhibitors (PIs), telaprevir and boceprevir, to pegylated interferon (PEG-IFN) and ribavirin (RBV) combination therapy significantly increased sustained virologic response (SVR) rates, PI-based triple therapy for the treatment of chronic hepatitis C virus (HCV) infection was prone to the emergence of resistant viral variants. Meanwhile, multiple direct acting antiviral agents (DAAs) targeting either the HCV NS3/4A protease, NS5A or NS5B polymerase have been approved and these have varying potencies and distinct propensities to provoke resistance. The pre-clinical *in vivo* assessment of drug efficacy and resistant variant emergence underwent a great evolution over the last decade. This field had long been hampered by the lack of suitable small animal models that robustly support the entire HCV life cycle. In particular, chimeric mice with humanized livers (humanized mice) and chimpanzees have been instrumental for studying HCV inhibitors and the evolution of drug resistance. In this review, we present the different *in vivo* HCV infection models and discuss their applicability to assess HCV therapy response and emergence of resistant variants.

## 1. Introduction

**Figure 1:**
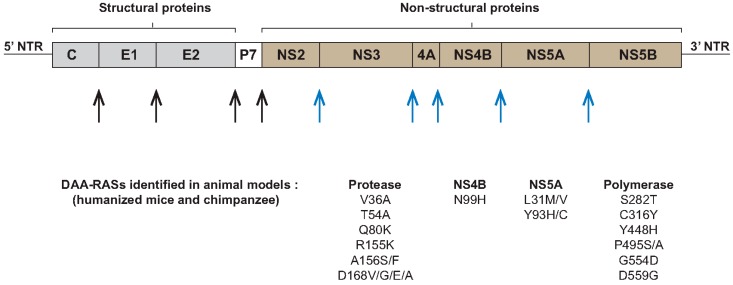
Hepatitis C virus (HCV) genome structure and direct acting antiviral agents (DAAs)-resistance associated substitutions (RASs). After viral entry in the hepatocyte the genome of HCV is translated into a single polyprotein precursor which is cleaved by host peptidases (black arrows) to release the structural proteins (C, E1 and E2) followed by cleavage by viral proteases (blue arrows) to produce the non-structural proteins (NS2, NS3, NS4A, NS4B, NS5A and NS5B). The NS3/4A and NS5B function as viral protease and polymerase, respectively. Mutations associated with resistance to different protease, polymerase and NS5A inhibitors that were identified during studies in HCV animal models are listed. NTR: non-translated region.

The hepatitis C virus (HCV) was identified in 1989 as the etiologic agent for non-A non-B hepatitis [1]. It is a major health problem with more than 170 million people infected worldwide. Approximately 80% of infected patients evolve to chronicity, which can ultimately progress to liver cirrhosis and hepatocellular carcinoma (HCC) [2]. HCV is a small enveloped positive-stranded RNA virus that belongs to the *Hepacivirus* genus and the *Flaviviridae* family. It is classified into seven genotypes and multiple different subtypes, which exhibit differences in pathogenesis and response to treatment. HCV of genotype 1 is the most common genotype worldwide and in developed nations [3]. Once HCV enters its host cell, the hepatocyte, the viral genome is translated into a polyprotein precursor that is cleaved into a set of structural and non-structural proteins. While this cleavage process is initiated by host peptidases that release the structural proteins of the virus, the non-structural proteins are cleaved by viral cysteine and serine proteases (Figure 1). The structural proteins include the core protein that forms the capsid of the virus, and the envelope glycoproteins E1 and E2. These envelope proteins interact with a variety of specific and non-specific host membrane proteins (attachment factors and (co-)receptors) to initiate viral entry. Two of these receptors, CD81 and occluding, play a key role in determining host cell and species tropism [4,5,6,7,8,9]. The P7 protein is involved in ion channel activity and viral assembly [10,11]. The non-structural proteins include NS2, NS3, NS4A, NS4B, NS5A and NS5B. NS3/4A and NS5B function as viral protease and RNA dependent RNA polymerase (RdRp), respectively, and are evidently required for viral replication. The NS4B and NS5A proteins are involved in the formation of membranous web but NS5A also plays a major role in orchestrating viral replication and assembly [11].

The high replication rate (about 10^12^ newly produced virions per day) and the error-prone HCV polymerase (10^−3^–10^−4^ substitutions per nucleotide per genome replication round) result in a cloud of closely related viral variants, known as quasispecies [12,13]. Within the quasispecies pool, variants will emerge that may be less sensitive to antiviral agents and which will become dominant within the population during drug pressure. A variety of non-structural proteins are the target of direct acting antiviral agents (DAAs). These include NS3-4A protease inhibitors, NS5A inhibitors and (non-)nucleos(t)ide NS5B polymerase inhibitors that possess distinct efficacy and genetic barrier to resistance. In addition, viral entry can be prevented by specifically blocking the envelope glycoproteins E1 and E2 or, in general, by targeting the viral particle with neutralizing antibodies or small molecules [14,15,16,17,18,19,20,21]. Besides viral proteins, also host cell factors that are involved in the HCV life cycle such as CD81, scavenger receptor class B type I (SR-BI), claudin-1, microRNA-122 (miR-122) and cyclophilin A can be antagonized as HCV treatment [22,23,24,25,26,27,28,29].

A 48-week combination therapy with pegylated interferon (PEG-IFN) and ribavirin (RBV) has long been the standard treatment protocol for chronic HCV infection. Besides low sustained virologic response (SVR), especially for some genotypes and people with certain genetic factors, the treatment was also accompanied by considerable side effects [30,31]. Telaprevir and boceprevir, in combination with PEG-IFN and RBV, were the first approved DAAs for HCV treatment [32]. Although this triple therapy improved response rates, especially for those patients infected with a genotype 1 virus and treatment-experienced patients, adverse effects including neutropenia, pruritus, anemia and thrombocytopenia have been reported at higher frequency than for the double therapy protocol [33]. In addition, because of their low genetic barrier to resistance, the triple therapy is usually accompanied by the emergence of resistant variants [34,35]. The preclinical evaluation of HCV drugs and their associated resistance had long been hampered by the lack of a robust small animal model for HCV infection. In this review, we give an overview of the currently available HCV *in vivo* models with an emphasis on their utility to study drug resistance.

## 2. Pre-Clinical in Vivo Models for HCV Treatment

### 2.1. Non-Rodent HCV Models

The experimental infection of chimpanzees (*Pan troglodytes*) enabled the discovery and identification of HCV as the etiologic agent for non-A non-B viral hepatitis [1,36]. It has been valuable for deciphering host-virus interactions and preclinical analysis of antiviral strategies [37]. Programmed cell death 1 (PD-1) and miR-122 antagonizing therapies are only two such examples [27,38]. Despite the genomic homology between chimpanzees and humans, the natural course of infection differs since only few chimpanzees develop chronic HCV infection, no fibrosis and only one hepatocellular carcinoma (HCC) case has been observed [39]. In the context of antiviral efficacy studies, chimpanzees have been successfully used to track resistance associated with the use of entry [40], protease, NS5A [41] and polymerase [42,43] inhibitors, and their combinations [44]. Nonetheless, availability, cost, and, particularly, ethical constraints severely limit the use of these large primates for HCV research. Recently, the National Institutes of Health (NIH) of the United States Department of Health and Human Services decided to effectively end its support for invasive research on chimpanzees [45].

Tree shrews (*Tupaia belangeri*) are non-rodent squirrel-like mammals that were found to be permissive for HCV infection [46,47]. While HCV viremia could only be detected intermittently, some signs of histological progression to HCV-related liver disorders were observed [48]. Although tree shrews have been used to evaluate hepato-protective activity against HCV-induced liver damage, compatibility of the *Tupaia* host environment with HCV replication seems rather low.

### 2.2. Rodent HCV Models

Due to the narrow host tropism of HCV, the development of practical small animal models for HCV, e.g., laboratory mice and rats, has been challenging [49]. Whilst rodents are naturally resistant to HCV infection different approaches have been undertaken to enable the study of the virus in mice. Of note, studies in HCV transgenic mice that express different, or a set of, viral proteins [50,51,52,53,54,55] have provided some insights in HCV pathogenesis but are less suited for screening HCV inhibitors. However, to productively match a human hepatotropic pathogen with a non-human liver, either the virus or the host should be adapted.

Although ongoing efforts are being made trying to adapt the virus to complete its life cycle in murine hepatocytes [56,57], no productive *in vivo* HCV infection has yet been reported in the absence of any human cofactor. One complementing strategy exists of transgenically supplementing mouse hepatocytes with essential human co-factors that would more efficiently support the HCV life cycle. Accordingly, adenoviral delivery of the human restriction factors CD81 and occludin (OCLN) allowed cell culture-derived HCV (HCVcc) entry into the mouse hepatocytes and warranted evaluation of entry inhibitors and vaccine candidates [5,16,21,58]. Furthermore, mice transgenic for four human entry factors (4hEF) and deficient in several innate immune signaling pathways reproduced the HCV life cycle beyond viral entry, with infectious virus being recovered from the mouse serum [6]. Recently, infectivity of HCV infected patient serum was observed using a similar entry factor transgenic approach in mice with intact innate immune system, albeit with very low level of viremia [59]. Of note, a two-week telaprevir treatment successfully reduced viremia in these transgenic mice challenged with a JFH1-based inoculum.

The approach that led to the most extensively used HCV animal model, has been the xenotransplantation of a human-liver derived tissue in mice. Huh7 cells (*i.e.*, human hepatoma cells that form the basis of *in vitro* HCV systems) that harbor a luciferase-based HCV subgenomic replicon have been transplanted into SCID/Beige mice, which enabled the study of HCV replication inhibitors [60]. Huh7 cells have also been transplanted in immunocompetent rats after in utero toleration. Although these Huh7-transplanted animals were shown to produce HCV viremia exceeding 10^4^ copies/mL, no infection data is available on tolerized rats transplanted with primary human hepatocytes [61]. One model that uses primary human liver tissue is the Trimera mouse, where small human liver fragments are transplanted under the kidney capsule or ear pinna. Ex vivo HCV challenge before extrahepatic implantation resulted in detectable viremia maintained for about one month [62]. Antiviral treatments tested in this model are an HCV internal ribosomal entry site inhibitor and anti-HCV human monoclonal antibodies [62,63].

The first fully permissive murine model that supported long-term HCV infection was produced by intrasplenic injection of human hepatocytes into immune deficient mice with diseased liver, such as Alb-uPA/SCID and FRG mice (respectively, urokinase–type plasminogen activator transgenic severe combined immunodeficiency mice, and fumarylacetoacetate hydrolase-recombination activating gene 2-interleukin-2 receptor, common γ-chain knockout mice) [64,65,66,67,68]. Hepatotoxicity in the Alb-uPA/SCID mouse is induced by overexpression of the urokinase-type plasminogen activator protein, which is under the control of the mouse albumin promotor [69]. In the FRG model, deficiency of the fumarylacetoacetate hydrolase enzyme (FAH) leads to accumulation of tyrosine catabolites that are toxic to mouse hepatocytes. The tyrosine catabolism pathway can be blocked upstream of the toxic metabolites by oral administration of 2-(2-nitro-4-trifluoromethylbenzoyl)-1,3 cyclohexanedione (NTBC) [68]. This allows easy control and induction of the liver toxicity. Although these two recipient mouse lines were predominantly used for generating human-liver chimeric mice [70], susceptibility to HCV infection has also been shown in MUP-uPA and TK-NOG mice [71,72]. All these models are based on constitutive or inducible mouse hepatocyte death, and severely attenuated cellular rejection, providing a favorable niche for the transplanted human hepatocytes to engraft and repopulate the mouse liver. This process results in mice with a human chimeric liver that renders them highly susceptible for human hepatotropic pathogens, and hence enables the study of HCV biology and evaluation of different antiviral strategies [70].

HCV that is present in the blood of infected patients is associated with host lipoproteins and circulates in the form of lipoviral particles (LVPs). Besides their role in viral infectivity, lipoprotein incorporation plays a major role in viral escape from host neutralizing antibodies [73,74,75]. Using a model encompassing the entire viral life cycle would be of great importance for studying the biophysical properties of different HCV particles. Differences between HCV produced in cell culture and viral particles circulating in the blood of infected patients have been extensively described. Since cell cultures generally do not support infection of clinical HCV isolates, *in vivo* models represent a valuable tool for studying HCV isolated from clinical samples. Although chimpanzees have been used to determine the biophysical properties of HCV particles [76], the immune competence of such animals may influence to some extent the characteristics of HCV virions via the presence of HCV-specific antibodies. Interestingly, blood samples isolated from (uninfected) mice with humanized liver showed a human like lipoprotein profile [77]. Infectivity and lipoprotein incorporation in LVPs has been tested in HCVcc and mHCVcc (HCVcc passaged in humanized mice) using density gradient ultracentrifugation [78,79,80]. These studies showed a density profile of viral particles isolated from chimpanzees and humanized mice similar to that observed in humans, but different from that of particles produced in cell culture. This was further corroborated recently by Calattini *et al*. who presented a comprehensive characterization of HCV particles isolated from humanized mice and cell culture [81]. The infectious HCV particles retrieved from mouse plasma displayed lower densities and high enrichment with apolipoprotein E, which has an impact on viral infectivity and receptor usage. Finally, mice humanized with both liver and immune cells are being developed that could provide new tools for studying immunopathogenesis, primary (human) adaptive immune response and vaccine strategies [82,83,84,85,86,87].

## 3. Principles of HCV Resistance

During the last few years, several DAAs including NS3/4A (protease), NS5A and NS5B (polymerase) inhibitors have been developed for HCV treatment [88,89,90,91]. The SVR achieved depends on several factors including the potency of the drug used and its genetic barrier to resistance, the regimen used, viral genotype/subtype and the presence/emergence of resistant variants. Since the viral polymerase lacks proofreading activity, closely related variants are continuously produced during the viral replication cycle. Most of resistance-associated substitutions (RASs) affect the viral fitness, especially those related to protease inhibitors (PIs), and hence could be found at low rates in treatment-naive patients [88,89,90,92]. Due to the selective pressure of DAAs, the wild type virus declines during therapy while resistant variants persist. Additional (compensatory) mutations are introduced and improve the viral fitness leading to viral breakthrough and treatment failure [89]. The possibility of viral relapse is related to different factors including the use of sub-optimal dose of the drug, a short duration therapy and the presence of resistant variants (possibly at low frequency below the limit of detection). Finally, the genetic barrier to resistance of the DAAs used plays a crucial role in success/failure of treatment. DAAs with a low barrier to resistance only need a few mutations (one or two substitutions) to confer resistance while multiple substitutions are required in case of DAAs with high barrier to resistance.

In the context of DAA-based therapy, several RASs including V36A/M, T54A/S, V55A, Q80K, R155K/T, A156T/S/V, D168A/E/T/V/N, V170A for PIs [32,89,90,93,94], M28T/V, Q30E/R/H, L31M/V, Y93H/C/N for NS5A inhibitors [90,95,96,97,98] and L159F, S282T, C316N, V321I/A, M426L, Y448H, Y452H R465G, V499A for polymerase inhibitors have been reported [99,100,101]. The resistance profile is genotype and subtype specific. For example, the PI-RASs V36M, T54S, R155K were reported in genotype 1a; V36A, T54S/A, A156S, V170A for genotype 1b; while the resistant variant D168Q is a natural polymorphism in genotype 3 [94,102]. Although resistant variants could be found at low frequency at baseline, they are dominant at treatment failure. A wide distribution of HCV-RASs in treatment-naive patients has been shown, including V36M, T54S, V55A, Q80K, R155K, V170A (NS3-RASs), L31M, Y93H as NS5A-RASs; and L159F, V321A/I, C316N, M426L, Y448H, Y452H, R465G, V499A, S556G as NS5B-RASs [91,99,101,103,104,105,106,107]. The impact of baseline RASs on treatment outcome depends on several factors including the type and frequency of RASs, the DAAs regimen used, the stage of liver disease, the prior DAAs treatment (treatment-experienced patients) and the virus genotype.

Treatment of HCV using a combination of two or more of DAAs has dramatically improved the SVR (> 90%). For example, the use of ledipasvir/sofosbuvir with or without RBV for genotype 1b treatment resulted in an SVR12 > 95%, although NS5A-RAS Y93H was detected at baseline [108]. Also, the use of ombitasvir/paritaprevir/ritonavir combination therapy for genotype 1b PEG-IFN/RBV-experienced patients resulted in > 88.9% SVR24 whereas a combination of the RASs NS3-D168V and NS5A-Y93H were found at the time of failure [109]. The use of dasabuvir/ombitasvir/paritaprevir/ritonavir with RBV for 12 weeks achieved SVR > 95% for genotypes 1a and 1b [110]. The most frequent variants found at the time of failure or relapse were D168V (NS3-RAS); M28T, Q30R, L31M, Y93H (NS5A-RASs); and S556G (NS5B-RAS) [110]. Recently, Pawlotsky provided a comprehensive overview on the different IFN-free DAAs regimens used for HCV treatment, concurrently proposing replacement of the term resistance associated variants (RAVs) by either “resistance associated substitution” (RAS) or “resistant variant” (RV) depending on whether reference is made to, respectively, the amino acid mutation that confers resistance or the resistant virus itself [89].

Interestingly, combination of daclatasvir with its analogue Syn-395 showed enhanced antiviral activity against NS5A resistant variants *in vitro* and in humanized mice [111]. This new finding paves the way for new regimens using DAAs from the same class that might improve the efficacy against resistant variants in treatment-experienced patients. Although the presence of RASs at baseline cannot predict treatment failure, screening at baseline for presence/frequency of those RASs leading to a high level of resistance is recommended (especially in DAAs-experienced and cirrhotic patients) in order to preclude the development of RV with multidrug resistance.

## 4. HCV Therapy Studies with Resistance Profiling in Human-Liver Mice

Besides chimpanzee, the human-liver chimeric mouse has been the only pre-clinical *in vivo* model used to monitor HCV drug resistance. As seen in the clinic, HCV treatment success is strongly dependent on the viral inoculum used to infect the animals. Besides genotype-specific treatment responses, multiple studies have confirmed expected decreased response rates after challenge with viral variants known to be resistant to the investigated drug. Indeed, pre-existing Q80K mutants in NS3, that confer partial resistance to PIs, were identified in both the inoculum and sera from mice that rebounded during BILN2061 (PI) therapy [112]. Virus from patient sera resistant to telaprevir (PI), mainly harboring the A156F mutation, was shown to be resistant to telaprevir treatment in mice as well [34]. Hiraga *et al*. went on to demonstrate that mice infected with an A156S mutant strain showed strong resistance to telaprevir treatment. Interestingly, the mutated virus replicated at lower rate than its wild type and showed high frequency of V36A during telaprevir therapy (11% at week 2) that decreased after treatment cessation (to 0.98%) before increasing again upon treatment re-initiation with higher dose (to 5.4% and 41.8% at week 1 and 4 respectively). Mice challenged with the wild type strain, however, first experienced rapid decline in viremia before telaprevir-resistant NS3 V36A variants started to emerge, suggesting that resistant HCV can be induced by mutation of the wild type strain *in vivo*. Another study from the same group confirmed that administration of telaprevir for four weeks in humanized mice results in a 2×log_10_-fold reduction in HCV viremia before viral rebound with emergence of the V36A mutation [34,113].

Further research performed by Chayama’s group was based on clonal infection (genotype 1b) confirming poor response to telaprevir of the NS3 V36A mutant, while remaining responsive to a sequential telaprevir-NS5A inhibitor (BMS-788329) combination therapy [114]. Then, they wanted to show the response of an NS5A-resistant variant (with RAS L31V) in a similar treatment schedule consisting of a sequential therapy of only BMS-788329 first, before addition of telaprevir to the therapeutic cocktail. While the L31V mutant was expected to be sufficient to confer resistance to BMS-788329, it only responded poorly upon accumulating an additional Y93C mutation. Under supplementary telaprevir pressure, this double mutant was able to accumulate a third mutation, resulting in the emergence of the NS5A/telaprevir-resistant NS5A L31V-Y93C/NS3 V36A variant. As expected, this triple-mutant emerged again under the same treatment conditions upon initial challenge with the NS5A double mutant (L31V-Y93C). However, in this case also the NS3 T54A mutant emerged. Replacing telaprevir by an NS5B inhibitor (BMS-821095) rapidly selected a fifth NS5B P495S mutation showing on-therapy breakthrough. In a similar therapeutic setup, although the initial challenge was done with the triple mutant (NS5A L31V-Y93C/NS3 V36A), the NS5B P495S variant rebounded after cessation of NS5A-NS5B inhibitor double therapy. Altogether, this study nicely highlights the issue of multi-drug resistant variant selection upon inappropriate sequential use of viral inhibitors. Very recently, mice challenged with HCV harboring the RASs: D168V, L31V and Y93H (isolated from a patient that failed asunaprevir (PI)/daclatasvir (NS5A-I) therapy) not only were resistant to this very same combination, but also to ledipasvir (NS5A-I)/GS-558093 (a NS5B inhibitor similar to sofosbuvir), while responding to telaprevir (PI)/GS-558093 therapy [115]. Most recently, the monotherapy of GSK9574, a prodrug for the NS4B inhibitor GSK8853, in HCV genotype 1a infected mice resulted in 3xlog_10_-fold decline in viremia with the emergence of the RASs N56I and N99H in NS4B [116].

Treatment of mice with the NS5B inhibitor MK-0608 resulted in an initial 2.6xlog_10_ decline in viremia before viral rebound and emergence of an NS5B S282T-containing resistant variant [113], similar to observations in chimpanzees [42,113]. Combination therapy of MK-0608 and telaprevir resulted in a decline of viremia to undetectable levels followed by viral rebound after cessation of therapy. However, no sequencing data is shown on these rebounding viral populations [113]. Of note, this combinatory regimen resulted in sustained virologic response upon addition of IFN-alpha or increased dosing of MK-0608. Together with a chimpanzee study [44], these were the first preclinical results suggesting that combination therapy using highly potent DAAs only, thus without IFN/RBV, is able to prevent resistance that could lead to viral rebound. Other pre-clinical evidence for the potential success of IFN/RBV free regimens using combination of different DAA classes is reported by Shi *et al*. [117]. While monotherapy of HCV genotype 1b infected mice with either the PI BMS-605339, NS5A inhibitor BMS-788329 or NS5B inhibitor BMS-821095 failed eradicating the virus due to resistant variants emergence during treatment (RASs D168E, Y93H, and P495A/S respectively), the combination of the NS5A inhibitor with either the PI or NS5B inhibitor was sufficient [117]. Of note, the same therapy was not as successful in the context of genotype 2a or 2b infections, hence providing useful genotype-specific preclinical treatment outcome information.

Human-liver chimeric mice have also been challenged with core mutants. One study described the further selection of the R70Q core mutant upon PEG-IFN/RBV treatment in mice injected with a sample obtained from a patient on PEG-IFN/RBV treatment [118,119]. However, another study that investigated the impact of R70Q and L91M core substitutions on response to IFN treatment in mice did not observe decreased susceptibility [118,119]. In addition, the model has been used to test the effect of, and identify resistant variants selected during, alternative anti-HCV approaches. For example, synthetic hairpin-shaped RNAs that can degrade the virus’ RNA genome effectively reduced the viral load before slowly rebounding after therapy cessation [120]. A follow-up study confirmed that this viral rebound was indeed due to emergence of a virus whose genome was mutated in the sequences targeted by the RNAs [120,121].

Very recently, our group reported data indicating that inclusion of an entry inhibitor to the newest DAA combination therapies may further increase response rates by preventing therapeutic failure caused by on-therapy breakthrough of DAA-resistant variants [122]. We first confirmed that HCV infected human-liver mice receiving DAA-monotherapy rapidly experienced on-therapy viral breakthrough. Deep sequencing analysis identified the manifestation of drug-resistant mutants upon viral rebound. However, when an entry inhibitor was added to the antiviral regimen, not a single mouse experienced on-therapy viral breakthrough despite selection of resistant variants in some of these animals. We hypothesized that the intrahepatic spread of resistant variants selected during the DAA therapy was efficiently blocked by the entry inhibitor [122].

**Figure 2:**
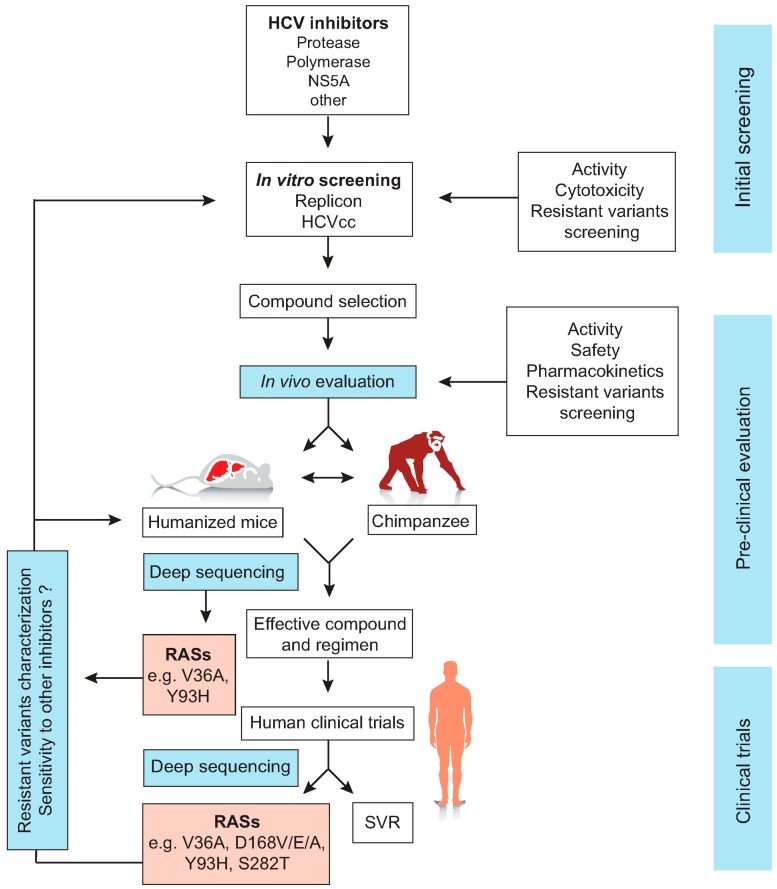
The sequence of HCV treatment and screening for resistance associated substitutions (RASs) and resistant variants. HCV protease, polymerase and NS5A inhibitors are screened first using HCV cell culture systems including HCV replicons and HCVcc models. Promising compounds with high potency and lowest cytotoxicity are then evaluated *in vivo* using the human liver chimeric mice and/or chimpanzee to test the pharmacokinetics, activity, safety and the emergence of resistant variants. Compounds with highest antiviral activity *in vitro* and *in vivo* could be then used in human clinical trials. If RASs are detected during clinical studies, the resistant variants could be further studied *in vitro* using replicons or HCVcc systems and in vivo using humanized mice or the chimpanzee. Such additional characterization studies would allow the evaluation of their sensitivity to other viral inhibitors. During therapy, the development of RASs could be identified using the clonal or deep sequencing (adapted from de Jong *et al*. 2011) [136].

Although the failure of DAA treatment is potentially related to the development of resistant variants during therapy, clonal and deep sequencing analyses revealed the presence of certain RASs at baseline. This might increase the risk of therapy failure upon treatment with different protease [123,124,125,126,127], polymerase [99,125,127,128,129,130] or NS5A inhibitors [95,99,127,131]. While most protease inhibitor resistant variants have reduced fitness compared to the wild type virus and hence rapidly disappear after cessation of therapy, other variants especially with mutations in NS5A (e.g., L31M and Y93H) can persist for a long time. Recently, ultra-deep sequencing showed that failure of asunaprevir and daclatasvir combination therapy in chronic patients might be associated with the emergence of the RASs L31M, Y93H and D168A/V variants [132,133]. In a study involving mice infected with genotype 1b quasispecies pool comprising PI-resistant virus, response to treatment with asunaprevir alone or in combination with daclatasvir correlated with the baseline frequency of these resistant variants [134]. In this study, resistant variants with RASs at NS3-D168 replaced the wild type virus upon administration of simeprevir. Inoculation of naive mice with these variants resulted in cross-resistance to asunaprevir, especially in mice in which the frequency of the RV was high. Similarly, the presence of D168 RASs at baseline resulted in a rebound of viremia after combination therapy of asunaprevir and daclatasvir [134]. Most recently, combination therapy of the PI vaniprevir and the NS5A inhibitor BMS-788329 eradicated HCV in genotype 1b chronically infected mice compared to viral breakthrough seen in mice treated with vaniprevir alone, especially in those mice which had high viremia [135]. Deep sequencing showed the presence of the RASs D168V/G/A in mice with vaniprevir treatment failure [135]. The above-mentioned studies clearly present human liver chimeric mice as a valuable model for the detailed study of treatment-associated resistance. Such studies could help in identifying new highly potent inhibitors with a high barrier to resistance or for the prediction of the optimal DAA treatment combination that can be effectively used against a viral quasispecies containing single or multiple RVs (Figure 2).

## 5. Conclusions

With the advent of deep-sequencing technology, which can detect mutants at much lower frequency than before, mutational analysis is becoming more and more available. The greatly improved sensitivity over techniques such as clonal and population sequencing is also particularly valuable in extracting the maximum possible information from limited sample size inherent to the use of small animal model systems. HCV therapy studies performed in chimpanzees and human-liver chimeric mice prove the relevance of these models in the context of antiviral resistance and confirm drug class-specific resistance profiles observed in cell culture systems and clinical trials. Either the animals responded less well to therapy after being challenged with virus that already harbored known RASs (clinical isolates or genetically engineered), or these mutants could be readily detected upon sequencing of on-therapy breakthrough or post-therapy relapse viral populations. We therefore believe that these animal models nicely bridge the gap between *in vitro* HCV systems and clinical trials. Of note, we have recently shown that human-liver mice challenged with HCV variants that show increased resistance to SR-BI blockade *in vitro*, can still be fully protected from infection by an anti-SR-BI mAb [79]. This highlights discrepancies that still exist between cell culture and animal systems and advocates that interpretation and extrapolation of *in vitro* findings should be done with caution.

Today, the most relevant preclinical small animal model for HCV therapy is the human-liver chimeric mouse. The Chayama’s group pioneered the use of such humanized mice for the analysis of resistant HCV mutants emerging during DAA therapy and for the validation of RASs identified in cell culture and clinical studies. They were among the first to provide pre-clinical evidence that DAA combinations of different classes, without IFN or RBV, were sufficient to reach sustained virologic response [113,117]. Their work also underscored that inappropriate sequential use of viral inhibitors efficiently selects for multi-drug resistant variants [114]. Recent work from our group in this model indicates that entry inhibitors can be used in combination with DAAs to prevent on-therapy breakthrough of DAA-resistant variants, thereby drastically improving end-of-treatment outcome [122]. Altogether, human-liver chimeric mice have been successfully used as a preclinical model to evaluate HCV therapeutics and monitor the development of resistant variants that are associated with their use (Figure 2). Considering the ethical constraints, limited availability and the high costs associated with chimpanzee studies, it is foreseeable that the human-liver chimeric mouse model will be the gold standard for preclinical *in vivo* evaluation of HCV treatment associated resistance.
